# Biodiversity assessment of tropical shelf eukaryotic communities via pelagic eDNA metabarcoding

**DOI:** 10.1002/ece3.5871

**Published:** 2019-12-03

**Authors:** Judith Bakker, Owen S. Wangensteen, Charles Baillie, Dayne Buddo, Demian D. Chapman, Austin J. Gallagher, Tristan L. Guttridge, Heidi Hertler, Stefano Mariani

**Affiliations:** ^1^ Department of Biological Sciences Florida International University Miami FL USA; ^2^ School of Engineering & Environment University of Salford Salford UK; ^3^ Norwegian College of Fishery Science UiT The Arctic University of Norway Tromsø Norway; ^4^ Discovery Bay Marine Laboratory and Field Station University of the West Indies St. Ann Jamaica; ^5^ Beneath the Waves Herndon VA USA; ^6^ Bimini Biological Field Station Foundation South Bimini Bahamas Bahamas; ^7^ The School for Field Studies Centre for Marine Resource Studies South Caicos Turks and Caicos Islands

**Keywords:** biomonitoring, Caribbean, ecosystems, environmental DNA, marine biodiversity

## Abstract

Our understanding of marine communities and their functions in an ecosystem relies on the ability to detect and monitor species distributions and abundances. Currently, the use of environmental DNA (eDNA) metabarcoding is increasingly being applied for the rapid assessment and monitoring of aquatic species. Most eDNA metabarcoding studies have either focussed on the simultaneous identification of a few specific taxa/groups or have been limited in geographical scope. Here, we employed eDNA metabarcoding to compare beta diversity patterns of complex pelagic marine communities in tropical coastal shelf habitats spanning the whole Caribbean Sea. We screened 68 water samples using a universal eukaryotic COI barcode region and detected highly diverse communities, which varied significantly among locations, and proved good descriptors of habitat type and environmental conditions. Less than 15% of eukaryotic taxa were assigned to metazoans, most DNA sequences belonged to a variety of planktonic “protists,” with over 50% of taxa unassigned at the phylum level, suggesting that the sampled communities host an astonishing amount of micro‐eukaryotic diversity yet undescribed or absent from COI reference databases. Although such a predominance of micro‐eukaryotes severely reduces the efficiency of universal COI markers to investigate vertebrate and other metazoans from aqueous eDNA, the study contributes to the advancement of rapid biomonitoring methods and brings us closer to a full inventory of extant marine biodiversity.

## Graphical abstract

Water samples from four Caribbean areas were sampled and subjected to universal eukaryote DNA metabarcoding. Tens of thousands of species were recovered, the vast majority being unicellular protists, most of which poorly known taxonomically and ecologically. Multivariate analyses clearly separated samples according to both region and habitat of origin.

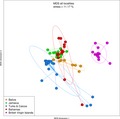

## 1 INTRODUCTION

Fundamental to ecosystem research and effective biodiversity management is the knowledge of which species are present in that ecosystem (Mace, Norris, & Fitter, 2012). Therefore, rapid assessment and monitoring of biodiversity are imperative, but the time and resources required to generate the necessary data are major constraints in ecology and conservation. Most recent estimates suggest that there are millions of marine eukaryotic species with the vast majority being small (<1 mm), cryptic, and currently unknown to science (Appeltans et al., 2012; Costello et al., 2012; Leray & Knowlton, 2016; Mora, Tittensor, Adl, Simpson, & Worm, 2011). Accordingly, there is a pressing need to measure marine biodiversity and to quantify the rate at which it is changing, particularly under adverse conditions such as overexploitation, climate change, and pollution. DNA‐based methods are revolutionizing the analysis of biodiversity, as they offer advantages over traditional, visual, and morphological survey methods (Thomsen &Willerslev, 2015). Accordingly, eDNA metabarcoding approaches have been successfully employed to characterize specific marine plankton communities in natural seawater samples, such as zooplankton, mesozooplankton, and full eukaryotic plankton diversity (Chain, Brown, MacIsaac, & Cristescu, 2016; Deagle, Clarke, Kitchener, Polanowski, & Davidson, 2017; de Vargas et al., 2015; Djurhuus et al., 2018; López‐escardó et al., 2018; Villarino et al., 2018), as well as benthic communities from both soft (Guardiola et al., 2015; Lejzerowicz et al., 2015; Pawlowski, Esling, Lejzerowicz, Cedhagen, & Wilding, 2014) and hard (Leray & Knowlton, 2015; Wangensteen, Cebrian, Palacín, & Turon, 2018; Wangensteen, Palacín, Guardiola, & Turon, 2018) bottom habitats.

There are many potential benefits that whole‐community metabarcoding of eukaryotic marine eDNA, using multiple or even single‐assay approaches, could bring to biodiversity assessment and monitoring, such as using direct measurements of biodiversity, instead of relying on biodiversity indicators (Aylagas, Borja, Irigoien, & Rodríguez‐Ezpeleta, 2016; Djurhuus et al., 2017; Lindenmayer & Likens, 2011; Rees, Maddison, Middleditch, Patmore, & Gough, 2014). Environmental DNA analysis can also be used for the detection of “hidden diversity,” without a priori knowledge of the composition of species assemblages in a particular water body (Boussarie et al., 2018; Lindeque, Parry, Harmer, Somerfield, & Atkinson, 2013). As such, eDNA can be a powerful tool in the early detection of alien species (Zaiko, Samuiloviene, Ardura, & Garcia‐Vazquez, 2015) and of community changes in response to environmental disturbances or regime shifts (Bik, Halanych, Sharma, & Thomas, 2012; Bucklin, Lindeque, Rodriguez‐Ezpeleta, Albaina, & Lehtiniemi, 2016). Gains in cost‐effectiveness, reproducibility, comprehensiveness, and the potential for multiple trophic levels to be evaluated simultaneously also make eDNA an attractive tool for large‐scale monitoring of biodiversity trends (Bourlat et al., 2013).

Studies targeting marine eukaryotic community diversity have, as of yet, been scarce (Cowart, Murphy, & Cheng, 2017; Deagle et al., 2017; Djurhuus et al., 2017; Drummond et al., 2015; Günther, Knebelsberger, Neumann, Laakmann, & Martínez Arbizu, 2018; Kelly, Gallego, & Jacobs‐Palmer, 2017; Villarino et al., 2018); the majority of these have relied on ribosomal markers, such as 18S (de Vargas et al., 2015; Guardiola et al., 2015; Lindeque et al., 2013), 16S (Kelly et al., 2016), and 12S (Miya et al., 2015), but due to their relatively conserved sequences, it is often impossible to distinguish taxa at the species, genus, or even family level (Tang et al., 2012), which represents a significant limitation to the evaluation of community changes and the biological and/or environmental mechanisms responsible for these changes (Mackas & Beaugrand, 2010).

Few recent studies have employed the mitochondrial cytochrome *c* oxidase subunit I (COI) marker region to offset the limited taxonomic resolution of ribosomal genes (Deagle et al., 2017; Lacoursière‐Roussel et al., 2018; Leray & Knowlton, 2017). The COI “barcode” region (Hebert, Ratnasingham, & deWaard, 2003) is one of the most commonly used DNA fragments for the analysis of species diversity among marine animals (Bucklin, Steinke, & Blanco‐Bercial, 2011). While the use of COI as a metabarcoding marker has been criticized, arguing that the high rates of sequence variability impair the design of truly universal primers and hamper the bioinformatic analysis (Deagle et al., 2014), the high mutation rate of COI may ensure unequivocal identification at the species level across a vast majority of taxa. Moreover, no other genetic region is currently represented in taxonomically verified databases to the same extent as the COI barcode region (Andújar, Arribas, Yu, Vogler, & Emerson, 2018).

Although no “gold‐standard” truly universal metabarcoding primer set has been identified for highly variable markers such as COI (Coissac, Riaz, & Puillandre, 2012; Deagle et al., 2014; Riaz et al., 2011), it has been shown that the taxonomic coverage and resolution provided by degenerate COI primers (primer sets that have one or more degenerate positions incorporated in either one or both of the forward and reverse primers), indeed make them valuable metabarcoding markers for biodiversity assessment (Clarke, Beard, Swadling, & Deagle, 2017; Elbrecht & Leese, 2016). Most recently, a degenerated version of the established COI internal primer set (Leray et al., 2013), amplifying a 313 bp region, has been described (Wangensteen, Palacín, et al., 2018). This primer set (henceforth referred to as “Leray‐XT”) features a high number of degenerate positions, including five deoxyinosine nucleotides (a nucleotide that complements any of the four natural bases) in the fully degenerated sites of the sequence, enhancing universality in the amplification of the COI fragment in most eukaryotic groups. Compared to 18S primers, these primers have been shown to reveal greater biodiversity at the species level when applied to the same samples (Wangensteen, Palacín, et al., 2018).

Even so, a number of questions remain unanswered: (a) What portions and segments of the total eukaryotic biodiversity present in tropical seawater is detectable through universal COI metabarcoding? (b) Is the method of using one universal metabarcoding marker powerful enough to distinguish between geographic regions and habitat types? (c) Are we close to introducing this approach to an applied, operational biomonitoring context? Here, in an attempt to answer these questions, we aimed to assess the potential for describing marine eukaryotic biodiversity from five distinct areas in the Caribbean, using the Leray‐XT primer set on eDNA obtained from taxonomically complex water samples. We evaluate the potential and scope of this primer set for the characterization of eukaryotic community structure, profiling of biodiversity, and for the assessment of spatial patterns between locations and habitats.

## 2 MATERIAL AND METHODS

### 2.1 Water sampling

**Figure 1:**
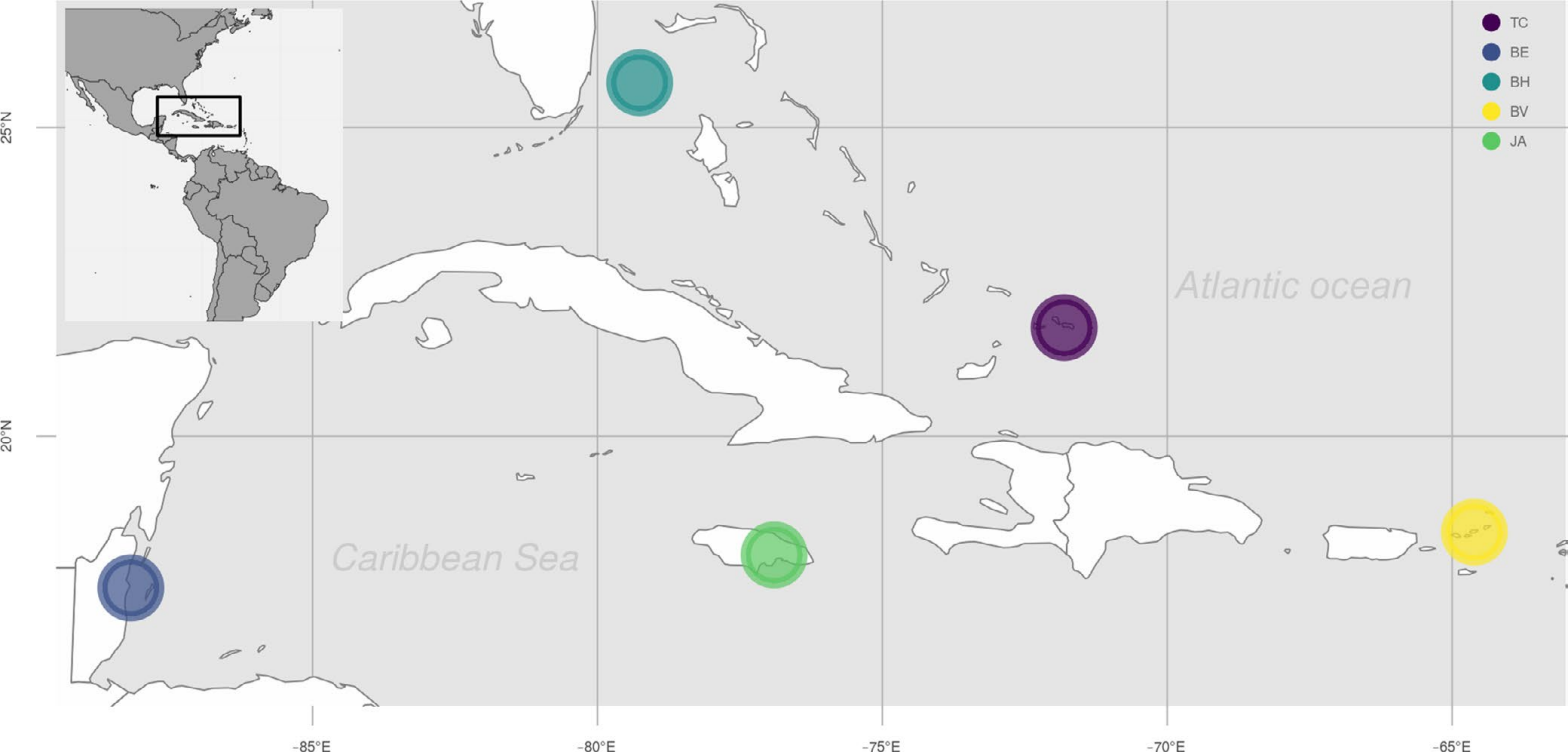
Map of Caribbean sampling locations. Bahamas, Belize, British Virgin Islands, Jamaica, and Turks and Caicos

Within each of five Caribbean sampling locations (Figure 1), samples were collected from different types of habitats in order to determine whether it would be possible to detect community differences on a smaller, local scale. In Belize, 8 samples were collected from the partially submerged Glover's Reef atoll, which is part of the Mesoamerican Barrier Reef. The sampling sites can be distinguished by either being back reef (shallow/inside the lagoon), or fore‐reef (deeper/towards open water) sites. In the Bahamas, 14 samples were collected around the islands of Bimini, from two distinct habitat types, mangroves and reef. Twelve samples were collected in Jamaica, where two specific sampling areas can be distinguished: Montego Bay and Discovery Bay. Montego Bay is a relatively large city, home to an airport and a cruise terminal, whereas Discovery Bay is a small town that receives much less tourism. In the Turks and Caicos Islands, 21 samples were collected from South Caicos, covering two main habitat types: mangroves and reef. In the British Virgin Islands, 13 samples were collected around Tortola, Virgin Gorda, and Eustatia Island, covering four distinct habitat types; bay, open reef, near‐shore reef and shallow shore sites. See Table S1 for full sampling specifics of all sites.

During February and March of 2015 (Jamaica, Belize, Turks and Caicos, and the Bahamas), and February and March 2017 (British Virgin Islands), a total of 68 water samples, of 4 L each, were collected with either a Kemmerer type water sampler, or, for practical reasons, directly with a disposable plastic collection bottle from sampling sites that were accessed by land, from the beach.

### 2.2 Sample processing and DNA extraction

After collection, water samples were individually covered and stored in the dark and on ice until further processing. Vacuum filtration was carried out within 2 hr of collection. The sterile mixed cellulose esters (MCE) filters (Merck Millipore; 47 mm diameter; 0.45 µm pore size) containing sample filtrates were stored in 2‐ml screw‐cap tubes containing silica beads. The silica beads function as a desiccator, drying out the filters and preventing the DNA from degrading (Bakker et al., 2017). The advantages of using silica beads instead of a liquid for DNA preservation include improved filter preservation, the prevention of leakages and the complications related to shipping/traveling with flammables (e.g., ethanol). The sample filters were subsequently stored, for approximately one month, at −20°C until extraction. DNA was extracted from the filters with the DNeasy PowerSoil DNA Isolation Kit (Qiagen), following the manufacturers' protocol. Purified extracts were assessed for DNA concentration in a Qubit fluorometer (Thermo Fisher Scientific).

### 2.3 Contamination control

Contamination of samples may occur anywhere from preparing sampling equipment and collecting the samples in the field (target DNA being carried unintentionally from one locality to another), to every subsequent step of sample preparation, extraction, and analysis in the laboratory. Hence, strict adherence to contamination control was followed at all field and laboratory stages in order to prevent the occurrence of contamination, including the use of disposable gloves and single‐use sterile collection bottles and filtration equipment, and the bleaching (50% bleach) of sampling devices and laboratory equipment and surfaces. Additionally, a dedicated controlled eDNA laboratory at the University of Salford, with separate rooms designated for the physical separation of eDNA extraction, pre‐PCR preparations, and post‐PCR procedures, was used for all laboratory work. Moreover, to identify potential contamination, negative field samples (filtration of drinking water purchased at local supermarkets), DNA extraction blanks (elution buffer from extraction kit), and PCR blanks (laboratory grade water) were included.

### 2.4 Library preparation and sequencing

We used a highly degenerated primer set, amplifying a 313‐bp segment from the COI region, which comprised the reverse primer jgHCO2198 5′‐TAIACYTCIGGRTGICCRAARAAYCA‐3′ (Geller, Meyer, Parker, & Hawk, 2013), and a modified forward primer mlCOIintF‐XT 5′‐GGWACWRGWTGRACWITITAYCCYCC‐3′ (Wangensteen, Palacín, et al., 2018). Samples were multiplexed by using primers with attached 8‐base sample‐specific oligo‐tags differing in at least 3 bases (Guardiola et al., 2015). In order to increase variability of the amplicon sequences, a variable number (2, 3, or 4) of fully degenerate positions (Ns) were added at the beginning of each primer (Wangensteen & Turon, 2017). The full, sequenced PCR product consisted of 389 bp, including the amplicon, primers, sample tags, and leading Ns. For PCR amplification, a one‐step protocol was used, which directly attached the 8‐base tagged primers in a single amplification. The mix recipe for this PCR included 10 μl AmpliTaq Gold DNA polymerase, 1 μl of each 5 μM forward and reverse primers, 0.16 μl bovine serum albumin (BSA), 5.84 μl sterile water, and a standardized amount (10 ng) of the filter‐extracted eDNA template, in a total volume of 20 μl per sample. The PCR profile included an initial denaturing step of 95°C for 10 min; 35 cycles of 94°C 1 min, 45°C 1 min, and 72°C 1 min; and a final extension step of 5 min at 72°C. The quality of all amplifications was assessed by electrophoresis, running the products through a 1.5% agarose gel stained with Gel Red (Cambridge Bioscience) and visualized on a UV light platform. All PCR products, including two extraction and two PCR negative controls, were pooled into one multiplexed sample and purified using the MinElute PCR Purification Kit (Qiagen). Two Illumina libraries were built on separate occasions, one containing the samples from the Bahamas, Belize, Jamaica, and Turks and Caicos, and one for the samples for the British Virgin Islands, as these samples were collected on a separate expedition. The library containing the British Virgin Islands samples was run alongside two other libraries (from an unrelated project), equalizing the sequencing depth across all samples by pooling an equal number of samples for each run. The libraries were built using the NextFlex PCR‐free library preparation kit (BIOO Scientific), quantified using the NEBNext qPCR quantification kit (New England Biolabs), and pooled along with 1% PhiX (v3; Illumina) serving as a positive sequencing quality control. The libraries, with a final molarity of 10 pM, were subsequently sequenced on an Illumina MiSeq platform, using v2 chemistry (2 × 250 bp paired‐ends).

### 2.5 Bioinformatic and statistical analysis

The bioinformatic analysis was based on the OBITools metabarcoding software suite (Boyer et al., 2016). The pipeline used for data analysis is summarized in Table S2. Paired‐end reads were aligned using illuminapairedend, retaining alignments with a quality score >30. The aligned dataset was demultiplexed, and primer sequences were removed with ngsfilter. A length filter (obigrep) was applied to the assigned reads in order to select only the fragments with the correct target size (300–320 bp). Reads containing ambiguous bases were also removed. The reads were subsequently dereplicated using obiuniq, grouping all the identical sequences, while keeping track of their abundances. A chimera removal step was performed using the uchime‐denovo algorithm (Edgar, Haas, Clemente, Quince, & Knight, 2011) implemented in vsearch (Rognes, Flouri, Nichols, Quince, & Mahé, 2016). Sequences were then clustered into Molecular Operational Taxonomic Units (MOTUs), in order to approximate the real species diversity in the samples. These MOTUs were delimited using the step‐by‐step aggregation clustering algorithm implemented in SWARM 2.0 (Mahé, Rognes, Quince, Vargas, & Dunthorn, 2015) with a *d*‐value of 13, which has been proven to be the optimal d‐value for the Leray fragment in different eukaryotic systems (Kemp et al., 2019; Siegenthaler et al., 2019). The SWARM 2.0 algorithm results in variable thresholds for delimiting MOTUs across different branches of the taxonomic tree, which is particularly pertinent with highly biodiverse samples such as those analyzed in this study. Taxonomic assignment of the representative sequences for each MOTU was performed using the ecotag algorithm (Boyer et al., 2016), which uses a custom reference database and a phylogenetic approach for assigning unmatched sequences to the last common ancestor of the most closely related sequences in the reference database. Ecotag infers a reference set of sequences including the best match, and other sequences present in the reference database, which are equally or more similar to the best match than the query sequence. Then, the query is assigned to the lowest rank taxon including all sequences in the reference set. As a result, taxonomy assignment by ecotag will yield different taxonomic ranks with different levels of uncertainty for different branches of the tree of life, depending on the density of the reference database for each branch. Thus, with each detected taxon, the percentage of identity with the reference sequence (%ID) is given as a measure of accuracy of the taxonomic identification. The custom COI reference database (db_COI_MBPK) contains 191.295 eukaryote sequences (Wangensteen, Palacín, et al., 2018), retrieved from the BOLD database (Ratnasingham & Hebert, 2007) and the EMBL repository (Kulikova, 2004), and is available from http://github.com/metabarpark/Reference-databases. After taxonomic assignment, putative pseudogene sequences were removed using LULU (Frøslev et al., 2017) to obtain reliable MOTU richness estimations. Finally, the dataset was refined by taxonomy clustering of MOTUs assigned to the same species, and minimal abundance filtering; all MOTUs with <2 reads were discarded.

All statistical analyses were performed in R v 3.3.0 (https://www.R-project.org/). Sample groups were represented in nMDS diagrams (function isoMDS) with Bray–Curtis dissimilarities, using the package MASS (Venables & Ripley, 2010). Using the adonis function in Vegan (v 2.5‐1; Oksanen et al., 2016), group distances between and within locations were formally tested with PERMANOVA, using fourth‐root transformed relative abundances. SIMPER analyses were also performed with the package Vegan, to identify the MOTUs that contribute most to the differentiation between the sampling locations.

## 3 RESULTS

### 3.1 Sequence read abundance and MOTU richness

A total number of 18,745,326 reads were obtained from the five sampling locations. After sample assignment, quality and sequence‐length filtering, removal of singletons, and removal of MOTUs assigned to bacteria or to the root of the tree of life, 2,391,157 reads were left from 68 samples. Between 14,884 and 55,608 filtered reads were generated per sample. Differences in the number of reads obtained per sampling site, among locations, were tested using ANOVA (*F* = 3.36, *df* = 4, *p* = .02) and pair‐wise Tukey's HSD. Pair‐wise comparisons showed that the mean number of reads varied significantly only between Jamaica and the Bahamas (diff = 10,390.55, *p*.adj = .03). Taxonomic assignment resulted in a total of 14,665 non‐singleton eukaryotic MOTUs, of which 6,357 (43.3%) could be assigned to phylum level or lower and 8,308 MOTUs (56.7%) remained unassigned.

Furthermore, LULU detected a total of 1,896 MOTUs putatively originating from pseudogenes, out of the total of 14,665 MOTUs. Thus, the number of corrected MOTUs in the final dataset is 12,769 (41.8% assigned to phylum level or lower and 58.2% remained unassigned). Rarefaction plots for individual samples from the five different areas (Figure S1) show that saturation in the total number of MOTUs is not achieved with this sequencing depth for most samples, indicating that the true extent of the MOTU richness assessed using this method may be higher than the values obtained in this study.

The negative controls contained a negligible number of reads, all of which were classified as human DNA, and hence were excluded from further analyses. Table 1 displays a summary of read statistics per location.

**Table 1:** Summary of the read statistics for each location

Location	Number of samples	Total *n* reads	Unassigned reads (%)	Metazoa reads (%)	Chordata reads (%)	Fish reads (%)	Total *n* MOTUs	Metazoa MOTUs (%)	Chordata MOTUs (%)	Fish MOTUs (%)
Belize	8	241,207	60.4	9.06	1.29	0.03	2,511	17.2	1.6	0.8
Bahamas	14	430,024	55.8	7.35	0.23	0.09	4,120	19.6	1.0	0.6
Jamaica	12	493,270	47.3	11.4	0.11	0.004	4,359	18.2	0.7	0.2
Turks & Caicos	21	724,164	70.8	13.9	0.83	0.06	6,774	23.0	0.7	0.4
BVI	13	502,492	52.3	14.9	0.07	0.008	5,203	23.0	1.0	0.4
Mean	13.6	478,231	57.3	11.32	0.51	0.038	4,593	20.2	1.0	0.48

Note

“Unassigned reads” are classified as “no taxonomic assignment (at any level) available”. Percentages of unassigned Metazoa, Chordata, and Fish reads are based on the total number of reads for each location. Percentages of Metazoa, Chordata, and Fish MOTUs are based on the total number of MOTUs for each location.

**Figure 2:**
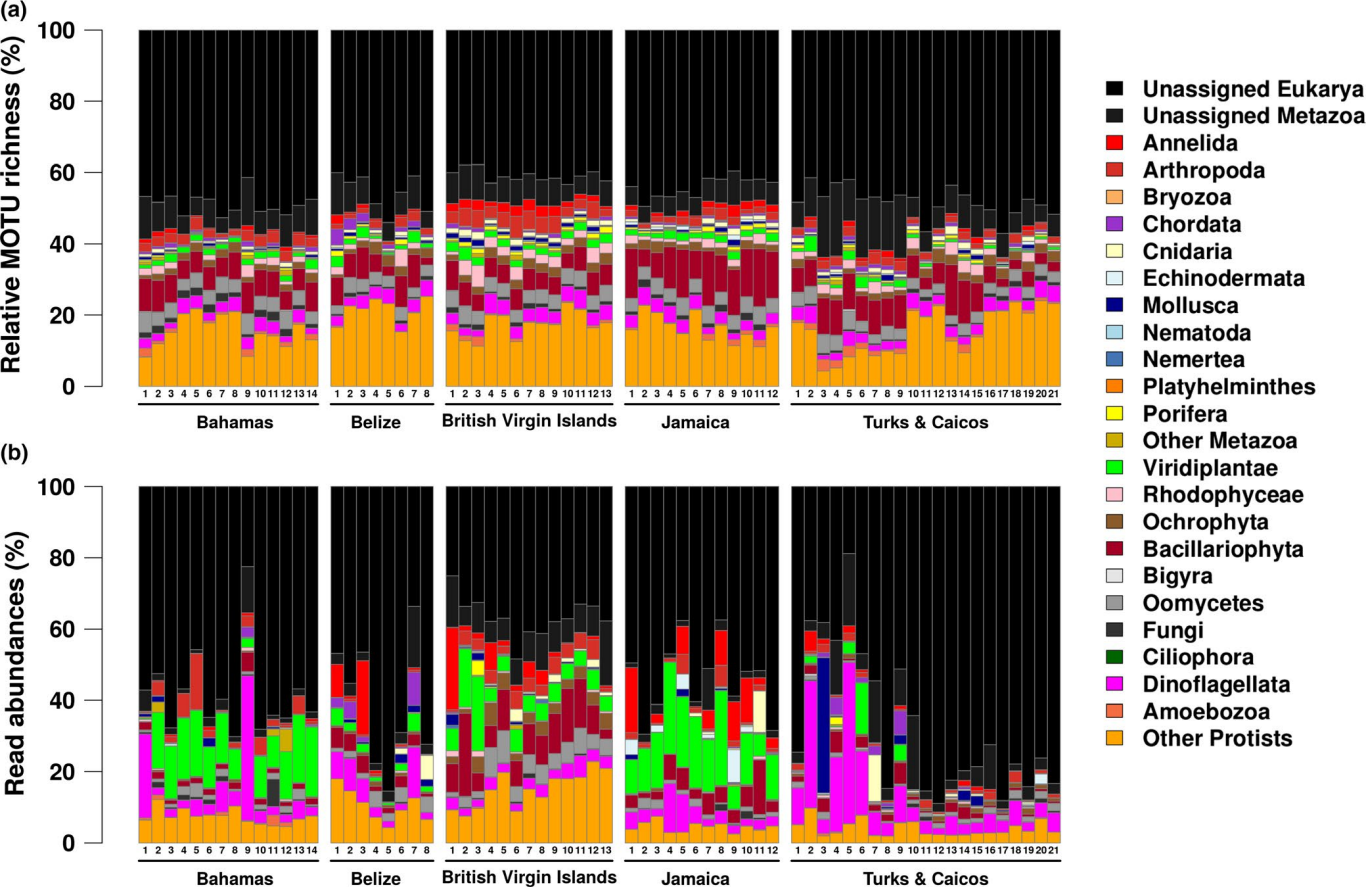
(a) Relative MOTU richness; the relative number of MOTUs assigned to the different phyla per sample, per location, and (b) relative number of reads of each of the different phyla, per sample, per location

The MOTUs assigned to major eukaryotic groups, for all the samples in each location, are presented in Figure 2a. The broad relative MOTU richness patterns across all five Caribbean locations are strikingly similar. The “unassigned Eukarya” group represents by far the largest relative MOTU richness in every sample, indicating the presence of a significant amount of eukaryotic biodiversity, which is either yet undescribed or not present in COI reference databases. Sequences assigned to protists make up the second most diverse group. Bacillariophyta (diatoms) and Dinoflagellata show relatively high relative diversity across all samples, as do the phyla grouped in the “other protists” category (orange bars at the bottom of Figure 2a), which include Apusozoa, Apicomplexa, Excavata, Hacrobia, Rhizaria, as well as the Alveolata, Stramenopiles, and Opisthokonta that could not be assigned to specific phyla. Macroscopic metazoans, such as Arthropoda and Annelida, additionally contain a relatively high MOTU richness. Sequences from Chordata are detected in most samples in four out of the five locations (with the exception of Jamaica, where only three samples contain chordate reads), but the number of MOTUs assigned to this phylum is relatively small.

The relative abundances of reads assigned to the different phyla in each sample are shown in Figure 2b. While the “unassigned Eukarya” remain the dominant component, read abundances show greater differentiation among samples and locations compared to the patterns of relative MOTU richness in Figure 2a.

By comparing relative MOTU richness and relative read abundances from different phyla, it is apparent that, for example, Bacillariophyta (diatoms) have a relatively high diversity in most of the samples, but reads assigned to this phylum are less abundant. An exception is the samples from the British Virgin Islands (BVI), and to some extent Jamaica, where Bacillariophyta reads are rather abundant (over 10% of all reads), mainly due to the preponderant abundance of *Rhizosolenia* (nearly 7% of all reads), a taxon that is detected only very sparsely in the other four locations (see https://doi.org/10.5061/dryad.hqbzkh1bj for all the reads per MOTU, per sample).

MOTU richness and read abundances for Oomycetes, common parasites on marine algae (Li, Zhang, Tang, & Wang, 2010), follow a similar pattern. Although a large part of the Oomycetes reads is assigned to MOTUs that only occur in the BVI samples, these could not be taxonomically assigned to any species, genus, or family. Additionally, the bulk of unidentified brown algae reads (Phaeophyceae) has been detected in this location. Other protist MOTUs that characterize the BVI samples are the cercozoan *Bigelowiella natans* (98%ID) and four marine centric diatoms: *Thalassiosira pseudonana* (100%ID), *Ditylum* (89%ID), *Grammonema* (87%ID), and *Skeletonema menzellii* (100%ID).

Green algae (Viridiplantae) generally exhibit lower diversity compared to other protist groups. However, this group's read abundances are particularly high in most of the samples from Jamaica and the Bahamas, and to a lesser extent in the BVI, with (in all three locations) most of the reads being assigned to either *Micromonas commoda* (99%ID) or *M. pusilla* (78%ID). Another significant portion of the green algae reads are assigned to *Mantoniella squamata* (93%ID) and/or *Dolichomastix tenuilepis* (92%ID). Viridiplantae reads are absent from most of the Turks and Caicos samples, where Dinoflagellata reads are more dominant. Overall, there appears to be a general pattern, both location‐wide, and per sample, whereby high abundance of Dinoflagellata generally corresponds to a low abundance of either green algae or diatoms, and vice versa. Most of the dinoflagellate reads could not be assigned beyond the class Dinophyceae. But reads assigned to *Gymnodinium catenatum* (98%ID) were detected in all but six samples. In many of the samples, reads from the dinoflagellate genus *Symbiodinium* (100%ID), which encompasses the largest and most prevalent group of (coral‐associated) endosymbiotic dinoflagellates (zooxanthellae), are also present. The BVI is the only location that has a relatively large number of reads assigned to Ochrophyta, with the bulk of the reads originating from brown algae (Phaeophyceae), some of which could be assigned to species level, such as the sessile reef associated *Canistrocarpus cervicornis* (100%ID) and planktonic *Chattonella subsalsa* (99%ID).

Among the Metazoa, Annelida, and Arthropoda are the phyla with the greatest relative MOTU richness across most locations (except for Belize, Figure 2a), and also tend to be the most abundant in terms of reads (see green and red segments in the Krona plots in Figure S2), with Molluscs (i.e., T&C), Chordates (e.g., Belize), Echinoderms (i.e., Jamaica), and Cnidarians (abundant everywhere except from the Bahamas), also representing a significant component (Figure S2). The most abundant molluscan species (all sequences recovered from T&C) were the aglajid sea slug, *Chelidonura hirundinina* (98%ID), and the mangrove periwinkle, *Littoraria angulifera* (100%ID). The majority of Cnidarian reads in the water column (sample 7 in Turks and Caicos) is not from coral‐building species, but made up of reads from the Hydrozoan *Cunina fowleri* (80%ID), although some Scleractinean coral species, such as *Siderastrea radians* (100%ID), *Porites astreoides* (100%ID), and *Stephanocoenia michelinii* (99%ID), were also detected. The Echinoderm reads in sample 19 from Turks and Caicos are all assigned to the brittle star *Ophiocoma echinata* (100%ID). Echinodermata reads in the Jamaican samples are, in addition to *Ophiocoma echinata*, assigned to *Lytechinus variegatus* (100%ID; green sea urchin), *Holothuria impatiens* (100%ID; bottleneck sea cucumber), and *Actinopyga agassizi* (100%ID; five‐toothed sea cucumber).

Most of the annelid reads could only be assigned to unidentified Polychaete worms. Lastly, most of the reads assigned to Chordata are derived from ascidian tunicates. Some of which could be assigned to species level, such as *Botrylloides nigrum* (100%ID). The large number of chordate reads in Belize samples 3 and 7 and Turks and Caicos 4 and 9 is entirely made up of unidentified species from the tunicate order Enterogona. Most of the remainder of the chordate reads were assigned to Actinopterygii (780 reads) and Chondrichthyes (294 reads), most of which could be taxonomically assigned to species level; 54 teleost and 6 elasmobranch species were detected (https://doi.org/10.5061/dryad.hqbzkh1bj).

### 3.2 Community structure

**Figure 3:**
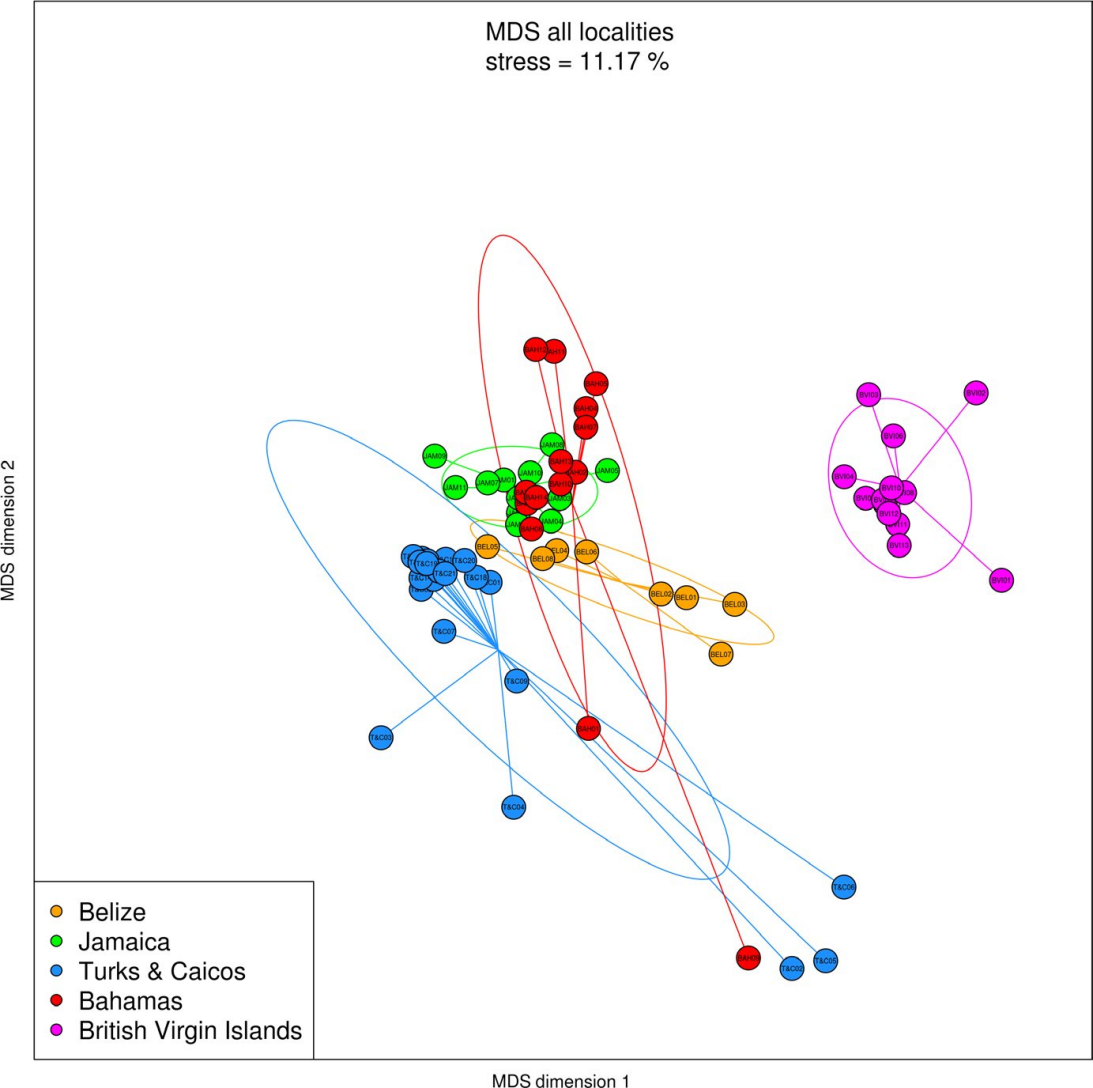
Non‐metric multidimensional scaling (nMDS) plot (Bray–Curtis, based on relative MOTU read abundances) showing the ordination pattern for the five sampling locations. Pair‐wise comparisons indicate significant differences in MOTU diversity between all the locations (*F* = 7.08, *df* = 4, *p* < .001)

The ordination of the sampling locations is visualized by a non‐metric multidimensional scaling (nMDS) plot (Figure 3). Pair‐wise comparisons indicate that significant differences in MOTU diversity exist among the five locations (*F* = 7.08, *df* = 4, *p* < .001). Turks and Caicos appears partially overlapped only with Bahamas; Belize and Jamaica exhibit a narrower range of variation and do not overlap with each other, but both partially overlap with some of the samples from Bahamas. The samples from the British Virgin Islands are completely separated from the other four locations. SIMPER (similarity percentage) analysis was applied to identify the discriminating taxa between the British Virgin Islands and the other four locations. The MOTU contributing most to this differentiation is assigned to an unidentified Eukaryotic taxon (MOTU number 703; responsible for 18.6% of the differentiation) that is abundant in the four overlapping locations but is very rare in the British Virgin Islands. SIMPER analysis results, consisting of a list of the 30 most discriminating phyla between the British Virgin Islands and the other four locations, can be found in Table 2.

**Table 2:** SIMPER analysis results identifying the 30 most discriminating taxa that combined contribute to 51.841% of the differentiation between the British Virgin Islands and the other four locations

	Rank	Scientific name	Super kingdom	Kingdom	Phylum	Average Bah, Bel, Jam & T&C	Average BVI	Cumulative sum
1	Superkingdom	Eukaryota (703)				31.82	0.944	18.601
2	Species	*Rhizosolenia setigera*	Chromalveolata	Stramenopiles	Bacillariophyta	0	6.27	22.361
3	Species	*Micromonas commoda*	Archaeplastida	Viridiplantae	Chlorophyta	5.366	2.208	25.838
4	Class	Dinophyceae	Chromalveolata	Alveolata	Dinoflagellata	6.146	1.577	28.624
5	Superkingdom	Eukaryota				2.093	5.048	31.044
6	Kingdom	Stramenopiles	Chromalveolata	Stramenopiles		0.007	3.443	33.105
7	Superkingdom	Eukaryota				3.018	0	34.915
8	Class	Oomycetes	Chromalveolata	Stramenopiles	Oomycota	0.816	3.156	36.506
9	No rank	*Micromonas pusilla*	Archaeplastida	Viridiplantae	Chlorophyta	0.877	2.863	38.012
10	Kingdom	Stramenopiles	Chromalveolata	Stramenopiles		0.605	2.546	39.242
11	Superkingdom	Eukaryota				1.73	2.292	40.192
12	Order	Spionida	Opisthokonta	Metazoa	Annelida	0	1.561	41.29
13	Kingdom	Stramenopiles	Chromalveolata	Stramenopiles		0.03	1.468	41.991
14	Kingdom	Metazoa	Opisthokonta	Metazoa		0.046	1.449	42.834
15	Kingdom	Metazoa	Opisthokonta	Metazoa		0.103	1.405	43.615
16	Superkingdom	Eukaryota				0.37	1.549	44.388
17	Order	Spionida	Opisthokonta	Metazoa	Annelida	0	1.046	45.015
18	Superkingdom	Eukaryota				0.041	1.037	45.617
19	No rank	Eumetazoa	Opisthokonta	Metazoa		0	0.989	46.21
20	Superkingdom	Eukaryota				0.094	1.051	46.792
21	Kingdom	Stramenopiles	Chromalveolata	Stramenopiles		1.115	0.824	47.37
22	Species	*Hematodinium *sp.	Chromalveolata	Alveolata	Dinoflagellata	0.029	0.955	47.925
23	Phylum	Bacillariophyta	Chromalveolata	Stramenopiles	Bacillariophyta	0.122	0.926	48.463
24	Kingdom	Stramenopiles	Chromalveolata	Stramenopiles		0.974	0.117	48.99
25	Superkingdom	Eukaryota				0.853	0.005	49.499
26	Superkingdom	Eukaryota				0.086	0.916	49.998
27	Superkingdom	Eukaryota				0.833	0.042	50.479
28	Superkingdom	Eukaryota				0.768	0	50.94
29	Phylum	Arthropoda	Opisthokonta	Metazoa	Arthropoda	0.152	0.755	51.397
30	Kingdom	Metazoa	Opisthokonta	Metazoa		0.013	0.754	51.841

Note

The MOTU contributing most to the differentiation is assigned to an unidentified Eukaryotic taxon, MOTU number 703, accounting for 18.601% of the differentiation. This MOTU is abundant in the four overlapping locations while rare in the British Virgin Islands.

In order to test whether the MOTU diversity in the British Virgin Islands would remain significantly different from the other locations without the abundant Eukaryotic MOTU 703, we verified that The British Virgin Islands ellipse remained fully separated from the other locations in the MDS (not shown) and pair‐wise comparisons remained significant between all locations (*F* = 7.02, *df* = 4, *p* < .001).

**Figure 4:**
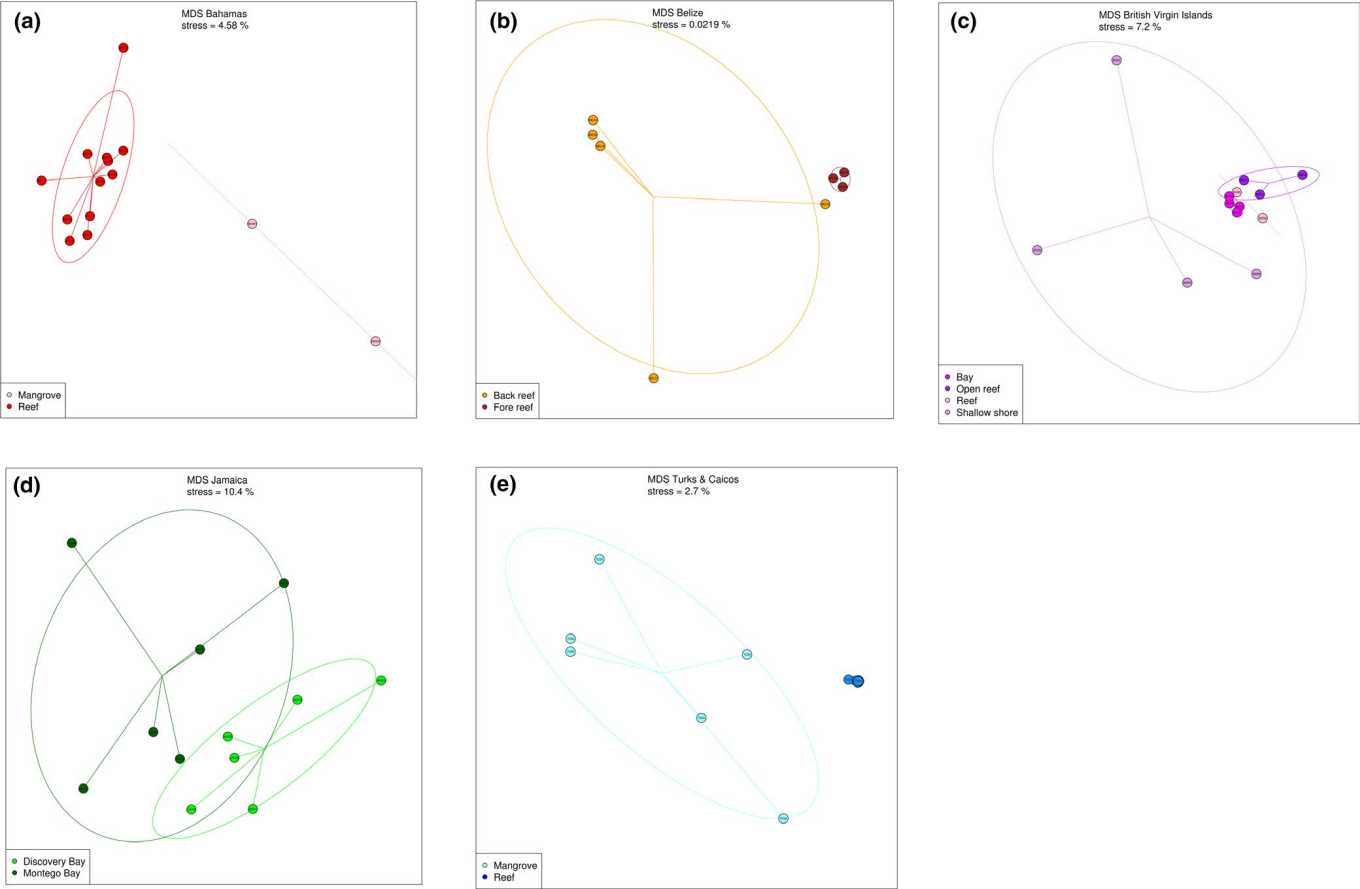
Non‐metric multidimensional scaling (nMDS) plots showing the ordination patterns within each of the five sampling locations

For each of the five sampling locations, individual nMDS plots are displayed in Figure 4, showing the ordination patterns within areas. In the Bahamas (Figure 4a), the two samples with the largest number of dinoflagellate reads (see also Figure 2b) are clearly separated from the rest (*F* = 5.22, *df* = 1, *p* < .01). These are samples number 01 and 09, which were collected from mangrove areas, while the other twelve samples originated from reef sites. SIMPER analysis of the Bahamian sampling site sequences (Data S1), indeed indicates that an unidentified dinoflagellate MOTU is a major contributor to the differentiation between the mangrove and reef samples, while the other two most differentiating MOTUs are an unidentified eukaryote MOTU and also the same eukaryotic MOTU 703 that is largely responsible for the separation between the British Virgin Islands and the other locations. Together with the green algae *Micromonas commoda* (99%ID), which is more abundant in the reef samples, these four MOTUs account for almost 37% of the differentiation between samples originating from reef sites and those from mangrove sites.

In Belize, samples were collected from both back‐ and fore‐reef sites, which is reflected in its ordination plot (Figure 4b). Here, the samples are converged into separate fore‐ and back‐reef ellipses (*F* = 3.54, *df* = 1, *p* < .05). The back‐reef samples are characterized by higher numbers of dinoflagellate, Bacillariophyta, Viridiplantae, and chordate (tunicate) reads, while the samples from the fore reef contain more “unassigned Eukarya,” Oomycetes and Cnidaria reads (Figure 2b). SIMPER analysis (Data S1) indicates that the unknown eukaryote MOTU 703 is responsible for almost 23% of the differentiation between the back‐ and fore‐reef samples, being more abundant in the fore‐reef sites. Additionally, the same unknown dinoflagellate sequences (sequence number 152) as in the Bahamas are the second most important MOTU, occurring more abundantly in the back‐reef samples. The additional differentiation within the back‐reef ellipse can be explained by the fact that these samples were collected from two different back‐reef sites (Table S1).

The sampling sites in the BVI are separated into four different groups (Figure 4c). The samples that were collected directly from the shore (without the use of a boat, *n* = 4) are most different from the samples that were collected from reef sites (*F* = 1.56, *df* = 3, *p* < .01). SIMPER analysis indicates that both the unknown eukaryote 703 and dinoflagellate MOTUs contribute most to the dissimilarity in MOTU diversity between the shore and the reef sites. Together with an unidentified mollusc MOTU, they contribute to almost 34% of the total dissimilarity (Data S1).

In Jamaica, samples were collected from two distinct sites, Discovery Bay and Montego Bay, which are grouped separately in Figure 4d. The unassigned eukaryote MOTU 703 (more abundant in Montego Bay), together with *Micromonas commoda* (99%ID) and an additional unassigned eukaryote MOTU, contribute almost 22% of the dissimilarity (*F* = 2.26, *df* = 1, *p* < .005) between the two sites.

In the Turks and Caicos samples, the two sampling environments (mangroves and reef) can be distinguished in the nMDS (Figure 4e). Similar to those from the Bahamas, the samples collected from mangrove areas are grouped separately from the samples collected from reef sites (*F* = 4.79, *df* = 2, *p* < .001). The unassigned eukaryote 703 is the most dominant MOTU in the reef samples and is responsible for almost 28% of the dissimilarity between the samples from the two environment types (Data S1). The unassigned dinoflagellate, being more abundant in the mangrove sites, represents the second most important MOTU. Moreover, Figure 3 reveals that the two mangrove samples from the Bahamas (samples 01 and 09), which are divergent from the Bahamian sample ellipse, tend to converge with the mangrove samples (02, 05, and 06) from Turks and Caicos. Within location, pair‐wise comparison results remain significant for all five locations after removing MOTU 703, Bahamas (*F* = 5.15, *df* = 1, *p* < .01), Belize (*F* = 3.56, *df* = 1, *p* < .05), BVI (*F* = 1.56, *df* = 3, *p* < .01), Jamaica (*F* = 2.25, *df* = 1, *p* < .005), and T&C (*F* = 4.73, *df* = 2, *p* < .001).

## 4 DISCUSSION

This study represents an exploration of the potential and scope of using a single universal primer assay for basin‐wide eukaryotic community eDNA screening of tropical coastal waters. Even though only ~11.200 species of marine eukaryotic plankton have so far been formally described (de Vargas et al., 2015). Our metabarcoding results from just across the Caribbean Sea show a high level of eukaryotic MOTU richness (12,769 MOTUs), which is mirrored in recent findings from similar locations, using similar tools (Nguyen et al., 2019). Additionally, we found that a disproportionally large amount of the reads (58.2%) could not be assigned to any further taxonomic level beyond Eukaryota.

Although this may in part be due to the incompleteness of DNA reference databases for COI, it suggests that the sampled communities may host a staggering amount of yet undescribed micro‐eukaryotic diversity. These results are in line with a recent study where 18S ribosomal DNA was used to assess eukaryotic diversity in photic‐zone plankton communities worldwide. It was estimated that eukaryotic ribosomal diversity saturated at ~150,000 operational taxonomic units, of which one‐third could not be assigned to known eukaryotic groups (de Vargas et al., 2015). As both the sample size and sequencing depth from de Vargas et al. (2015) are much larger compared to the present study, the spectrum of species diversity detected is also much greater (about 10 times). Additionally, instead of water samples, plankton nets were used to concentrate plankton density in each sample in order to recover complete local eukaryotic biodiversity (de Vargas et al., 2015). However, the 18S rDNA gene has limited taxonomic resolution power compared to COI used in this study (albeit with a smaller sample size). Therefore, it is very likely that for both studies, the level of species richness is still highly conservative, and could see a significant increase when a combination of high‐resolution primers and large sample size will be used.

Compared to more targeted metabarcoding applications, the marker used in this study allows coverage of the largest taxonomic breadth and the most powerful taxon delineation for any one marker. This results in the paradox of uncovering a substantial amount of diversity, much of which remains hidden. Irrespective of the level of identification, abundant, putatively ecologically important sequences are included in the overall biodiversity analysis, and the effect of these important taxa on β diversity may still be evaluated, even when these sequences cannot currently be taxonomically assigned to a known morphological group or species. Much of this diversity would remain undetectable if taxonomic assignment was solely based on a high percentage identity match. This is abundantly highlighted by the fact that SIMPER analyses (Data S1) suggest that currently unidentifiable MOTUs may actually be largely responsible for the differentiation of certain communities. Of those analyzed in this project, 50% of the differentiation between the different locations/communities is caused by less than 30 MOTUs, of which most have a best ID of <90% with a reference sequence present in the COI database. These dominant MOTUs would have been left out of any taxonomic assignment based on a high level of similarity and would likely have gone wholly undetected using traditional morphological approaches. Our data indicate that those taxa, which are a component of the currently unidentified diversity, are in fact most important both for differences in read abundances, and β‐diversity between locations and additionally, also between sites within these locations. A prime example is the unassigned eukaryote MOTU 703, a taxon that contributes notably to the differentiation between the sampled communities. While its removal from the analyses does not have a significant effect on the ordination patterns of neither the locations nor the sites within each location, this taxon is for example responsible for 27.5% of the variability between the mangrove and reef samples from Turks and Caicos, and it explains almost 23% of the variability between the fore‐ and back‐reef sites in Belize. The representative sequence of this MOTU is sequence number 703 to appear in the MOTU database, which indicates that it is highly abundant (608.941 reads) and is thus likely to be a relatively important component of the sampled communities. This novel sequence may either belong to a yet completely undescribed species, or to a species that has already been described morphologically and/or genetically identified with other markers, but for which the COI marker has yet to be sequenced. The ecological importance of this, and other undescribed MOTUs, to these areas is unknown but should not be overlooked.

A key group in our dataset that is defined by many unidentified MOTUs is the “Other Protists,” a typically diverse and heavily undersampled group (de Vargas et al., 2015; Foissner, 2008), that may display a wide range of trophic modes (Vaulot, Romari, & Not, 2002) and includes a high diversity of parasites and photosymbiotic taxa (de Vargas et al., 2015). Our dataset also contains a relatively high diversity of undescribed animal sequences, indicated by the extent of MOTU richness in the “Unassigned Metazoa” group (Figure 2a). It has previously been suggested that many animal lineages remain unsampled and/or unsequenced, potentially even harboring novel phyla (López‐escardó et al., 2018; Nguyen et al., 2019).

The British Virgin Islands were sampled two years after the other locations, which could potentially have played a role in the pronounced separation of this location compared to the other four. However, the samples were collected during the same season (February and March). Moreover, the MOTU explaining 18.6% of the variability between the British Virgin Islands and the other locations is the unknown eukaryotic MOTU 703, which is much less abundant here but was predominant elsewhere. Additionally, except for the Bacillariophyta Rhizosolenia (87%ID), most of the other important MOTUs in the British Virgin Islands samples were also detected in the other four locations. There is the possibility that inter‐annual fluctuations in the relative proportions of the most abundant taxa can cause substantial basin‐scale changes in microplankton communities, but the difference between and within areas should be robust to such changes, as shown by the significant distinction among samples from the same year. The biological relevance of the differences observed between the BVI and the other locations remains to be evaluated; an explicit temporal dimension should be formally considered in marine eDNA studies aimed at understanding community ecology (Lacoursière‐Roussel et al., 2018).

All sampling methods are subject to methodological limitations, and different sampling methods will capture different subsets of biodiversity (Kelly et al., 2017; Shelton et al., 2016). Like more traditional survey methods, eDNA metabarcoding has a certain level of taxonomic selectivity, which may be the result of primer bias. The use of COI as a metabarcoding marker has been criticized in the past (Deagle et al., 2014) owing to its high sequence variability, which may impair the design of truly universal primers and complicate bioinformatics analysis. However, the use of primers with high degeneration rates and including deoxyinosines has contributed to mitigate these universality issues (Wangensteen, Palacín, et al., 2018).

Additionally, when using COI as a metabarcoding marker, particularly when applied to samples containing highly complex signals, false‐negative detection errors are likely to occur. Conversely, the number of species (MOTUs) will often be overestimated due to the detection of pseudogenes, such as “numts,” nuclear sequences of mitochondrial origin (Bensasson, Zhang, Hartl, & Hewitt, 2001; Vamos, Elbrecht, & Leese, 2017), intraspecific variability (divergent haplogroups), or intra‐individual variability. Conversely, it can also be argued that COI presents two major advantages over other potential markers. First, the steadily growing international effort, headed by the Consortium for the Barcode of Life (CBOL), to develop a public DNA barcoding database with curated taxonomy, greatly facilitates taxonomic assignment. Secondly, the high mutation rate of COI enables identification at the species level, whereas the highly conserved sequences of other markers, such as 18S, make it often impossible to distinguish at the species or genus levels. Moreover, the combination of different metabarcoding markers within the same study, with different levels of taxonomic resolution and completeness of reference databases, may produce datasets displaying dissimilar community structure and prevent the use of relative read abundances as a quantitative measure of ecological importance. Consequently, if for this study we had chosen to use a primer set targeting a different marker, such as 18S rRNA (which is often used for studies of planktonic eukaryotes), the dominant unassigned eukaryote MOTU 703 might have been identified to a lower taxonomic level, while other taxa that were identified to species level by the Leray‐XT primers may not have been identified at all (de Vargas et al., 2015; López‐escardó et al., 2018), due to the limited taxonomic resolution power. Accordingly, recent studies have been adapting their strategy concerning the detection of community structure with single‐assay studies, and instead opt to include multiple assays to improve detection probability, in order to provide an improved approximation of eukaryotic community diversity, derived from eDNA samples (Djurhuus et al., 2018; Günther et al., 2018; Stat et al., 2017; Villarino et al., 2018). These studies aim to overcome the occurrence of false negatives due to primer bias, at the expense of the potential for quantitative inference, so that only presence/absence methods can be used in downstream analyses.

The vast majority of marine life is physically small and dominant in both numbers and diversity (Guil, 2011; Snelgrove, 1999). Consequently, in a water sample, eDNA from small eukaryotic organisms far outnumbers that of any vertebrate species, rendering the detection of eDNA from species such as teleosts in unfractionated water samples with a broad‐spectrum primer, similar to an eDNA needle in the proverbial haystack (Collins et al., 2019). Even more so, at least part of the DNA from microscopic eukaryotes will have originated from entire individuals, as opposed to exclusively extracellular DNA from larger species, potentially drowning out part of the eDNA signal from these larger organisms. Nonetheless, the Leray‐XT primer set was capable of detecting 54 teleost and 6 elasmobranch species. The use of a combination of multiple metabarcoding markers is essential to guarantee sufficient sequencing depth for capturing all the main components of eukaryotic diversity, including the better known metazoans groups upon which most conservation initiatives are based (Boussarie et al., 2018). Of course, the use of a single marker will significantly reduce operational costs, but a universal COI approach from aqueous eDNA samples, in the fashion of what is presented here, will always inevitably emphasize the micro‐eukaryotic component.

While biodiversity loss has been exhaustively documented for macro and mega fauna (which only represent a small fraction of total marine biodiversity), to our knowledge, no research has addressed specifically this issue pertaining to microscopic eukaryotic plankton communities, which is most likely due to the difficulties in characterizing and quantifying the diversity of these communities (Bouchet, Lozouet, Maestrati, & Heros, 2002; Fonseca et al., 2010; Hirai, Kuriyama, Ichikawa, Hidaka, & Tsuda, 2015). However, marine plankton is essential for biological and geochemical processes, fixing CO_2_ and other elements into biological matter, which subsequently enters the food web (de Vargas et al., 2015; Field, Behrenfeld, Randerson, & Falkowski, 1998; Pernice et al., 2016). As such, micro‐eukaryotic plankton plays essential roles in the structure and function of marine ecosystems globally (Liu et al., 2017). Additionally, planktonic communities are often used as indicators of ecosystem change due to their ability to rapidly respond to environmental shifts (Abad, Albaina, Aguirre, & Estonba, 2017; Taylor, Allen, & Clark, 2002). This makes the understanding of the composition, dynamics and position in food webs of planktonic communities across space and time, essential.

Interestingly, despite the thousands of taxa detected in our study, only tens of those were enough to distinguish samples at both regional and local scales, which suggests that in spite of the current major gaps in reference databases, COI metabarcoding of unfractionated water samples can be useful for describing coarse biodiversity trends, even when taxonomic assignment for key MOTUs is yet unavailable (Cordier et al., 2017). In fact, for certain ecological applications, a DNA‐based monitoring approach may be streamlined by using just a few “pelagic indicator” taxa, although such applications would require the formal description of such indicator species. Species‐level analysis of pelagic biodiversity is critical for understanding impacts of climate change, detecting invasive species, and the design of management objectives (Bucklin et al., 2016; Leray & Knowlton, 2016), hence the unraveling of the composition of hidden eukaryotic diversity and its subsequent description remain essential tasks, as comprehensive reference databases are critically needed for the taxonomic designation of eukaryotic DNA sequences. And while it has been estimated that between 24% and 98% of marine eukaryotic species are yet to be described (Goodwin et al., 2017; Leray & Knowlton, 2016; Mora et al., 2011), the advent of high‐throughput sequencing and DNA metabarcoding is rendering the huge task of uncovering this hidden diversity using a “reverse taxonomy” approach (Markmann & Tautz, 2005), and in particular taxonomic assignment with COI metabarcoding (applying either single or multi‐assay approaches), less insurmountable. Simultaneously, studies aimed at identifying areas harboring high numbers of potentially important micro‐eukaryotes could serve to direct targeted sampling to examine the most abundant microplankton, using powerful microscopy, in order to verify the identity of these taxa, which potentially hold great ecological importance.

As this study merely represents an initial exploration of the possibilities of using a COI metabarcoding assay for the description of marine eukaryotic community diversity, it by no means captures the full potential of eDNA metabarcoding for this purpose. However, it does clearly display the future possibilities and benefits of the method, underlining the importance of multiple assays as opposed to one single “universal” metabarcoding assay for the description of whole eukaryotic community assessment. Moreover, it particularly highlights the effect and significance of currently unidentified micro‐eukaryotic diversity on marine community structure and advocates for its inclusion in biodiversity assessments.

## CONFLICT OF INTEREST

None declared.

## AUTHOR CONTRIBUTIONS

JB and SM conceived the study. Samples were collected by JB, DB, DDC, AJG, TLG, and HH. JB and OSW carried out laboratory work and analyzed the data with CB. All authors interpreted and discussed the results. JB drafted the manuscript, with contributions from OSW and SM.

## Data Availability

Full sequence data can be found here: https://doi.org/10.5061/dryad.hqbzkh1bj.
