# Supplementary material for: Biodiversity assessment of tropical shelf eukaryotic communities via pelagic eDNA metabarcoding
Source: Ecol Evol. 2019 Dec 3;9(24):14341–55. doi: 10.1002/ece3.5871 (PMC6953649; doi:10.1002/ece3.5871)
Supplement: Supplementary file 2 [file ECE3-9-14341-s002.docx]

**Table S2. Summary of the bioinformatics pipeline used for COI Leray-XT marker. owi_recount_swarm, owi_add_taxonomy and owi_collapse are custom R scripts available from** [**http://github.com/metabarpark**](http://github.com/metabarpark)

| **Process** | **Software** |
| --- | --- |
| Raw sequences QC | fasttqc  fastx_trimmer |
| Paired-end alignment and  post-alignment QC | illuminapairedend  obiannotate  obisplit |
| Demultiplexing | ngsfilter |
| Length filter 300-320 bp | obigrep |
| Dereplication | obiuniq |
| Rename identifiers | obiannotate |
| Chimera removal | VSEARCH uchime_denovo |
| Clustering | SWARM v2 d=13  obitab  owi_recount_swarm  delete singletons |
| Taxonomic identification | ecotag using db_COI_Sep2017 |
| Add higher taxa | owi_add_taxonomy |
| Delete Prokaryota | Calc |
| Taxonomy clustering (collapse unique species) | owi_collapse |
| Final refinement | Blank correction  Minimal abundance filtering (>1 reads) |

**Ecotag_LULU pipeline**

#!/bin/bash

INPUT_FILE=SHOK_S1_L001_R1_001.fastq

LIBE="SHOK"

CORES=50

echo Unzipping fastq files.

gunzip *.gz

echo Splitting initial files in $CORES fragments.

obidistribute -n $CORES -p ''$LIBE"_R1_part" $INPUT_FILE

obidistribute -n $CORES -p ''$LIBE'_R2_part' ${INPUT_FILE//R1_001.fastq/R2_001.fastq}

echo Doing paired-end alignment, demultiplexing and length filter

for i in $(seq 1 1 $CORES) ; do illuminapairedend -r ${LIBE}_R2_part_$(printf "%02d" $i).fastq ${LIBE}_R1_part_$(printf "%02d" $i).fastq | obigrep -p 'score>40.00' | ngsfilter -t ngsfilter_COI_LerayXT_CEAB.tsv | obigrep -p 'seq_length>300' -p 'seq_length<320' -s '^[ACGT]+$' --fasta-output > LIBE.filtered_length_part_$(printf "%02d" $i).fasta & done

wait

cat LIBE.filtered_length_part*.fasta > $LIBE.joined.fasta

echo Getting stats file with sequencing depth per sample

obistat -c sample -a seq_length $LIBE.joined.fasta > sample_stats_$LIBE.txt &

echo Dereplicating unique sequences in a single file

obiuniq -m sample $LIBE.joined.fasta > $LIBE.unique.fasta

wait

echo Changing the identifier to a short index

obiannotate --seq-rank $LIBE.unique.fasta | obiannotate --set-identifier '"'$LIBE'_%09d" % seq_rank' > $LIBE.new.fasta

echo Converting to vsearch format

owi_obifasta2vsearch -i $LIBE.new.fasta -o $LIBE.vsearch.fasta

echo Removing Chimaeras in a single file

vsearch --uchime_denovo $LIBE.vsearch.fasta --sizeout --nonchimeras $LIBE.nonchimeras.fasta --chimeras $LIBE.chimeras.fasta --uchimeout $LIBE.uchimeout.txt

echo Obtaining the table file with abundances

obitab -o $LIBE.new.fasta > $LIBE.new.tab &

echo Clustering using SWARM

swarm -d 13 -z -t 40 -o $LIBE.SWARM13nc_output -s $LIBE.SWARM13nc_stats -w $LIBE.SWARM13nc_seeds.fasta $LIBE.nonchimeras.fasta

wait

echo Recounting after SWARM

owi_recount_swarm $LIBE.SWARM13nc_output $LIBE.new.tab

echo removing singletons

sed -i 's/;size/ size/g' $LIBE.SWARM13nc_seeds.fasta

obigrep -p 'size>1' $LIBE.SWARM13nc_seeds.fasta > $LIBE.seeds_nonsingleton.fasta

echo Splitting seeds file into $CORES pieces

obidistribute -n $CORES -p $LIBE.seeds $LIBE.seeds_nonsingleton.fasta

wait

echo Taxonomic assignment with ecotag

for i in $(seq 1 1 $CORES) ; do ecotag -d ~/taxo/taxo_Nov2018 -R ~/taxo/db_COI_Nov2018.fasta $LIBE.seeds_$(printf "%02d" $i).fasta > $LIBE.seeds.ecotag_$(printf "%02d" $i).fasta & done

wait

echo Adding higher taxonomy ranks

cat $LIBE.seeds.ecotag_??.fasta > $LIBE.ecotag.fasta

owi_add_taxonomy $LIBE.ecotag.fasta

echo Combine taxonomy and abundance files

owi_combine -i $LIBE.ecotag.fasta.annotated.csv -a $LIBE.SWARM13nc_output.counts.csv -o $LIBE.All_MOTUs.csv

echo Producing a pairwise match list to be used by LULU

vsearch --usearch_global $LIBE.seeds_nonsingleton.fasta --db $LIBE.seeds_nonsingleton.fasta --self --id .84 --iddef 1 --userout $LIBE_match_list.txt -userfields query+target+id --maxaccepts 0 --query_cov .9 --maxhits 10

echo Removing pseudogenes with LULU

Rscript $LIBE.LULU.R

echo Final file $LIBE.Curated_LULU.csv written

echo DONE!
