# Supplementary figures and images for: Biodiversity assessment of tropical shelf eukaryotic communities via pelagic eDNA metabarcoding
Source: Ecol Evol. 2019 Dec 3;9(24):14341–55. doi: 10.1002/ece3.5871 (PMC6953649; doi:10.1002/ece3.5871)

**Bahamas**

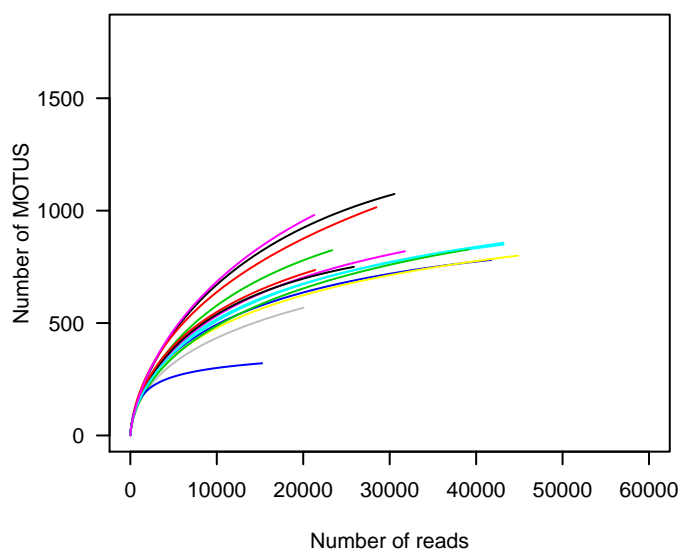

**Belize**

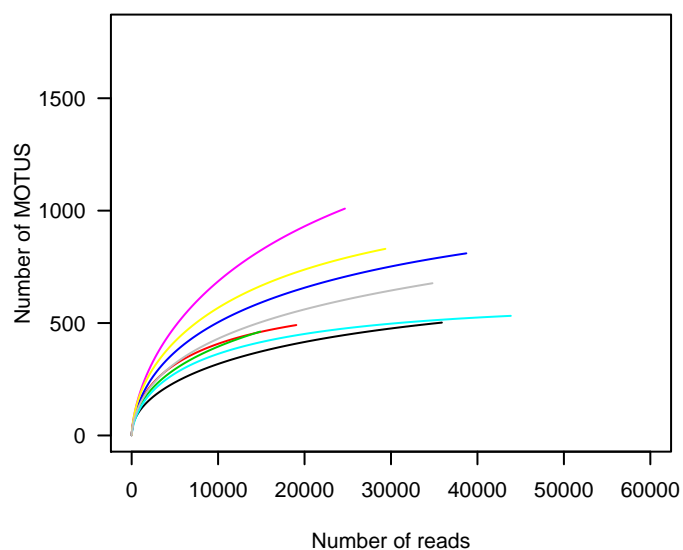

**British Virgin Is. COI**

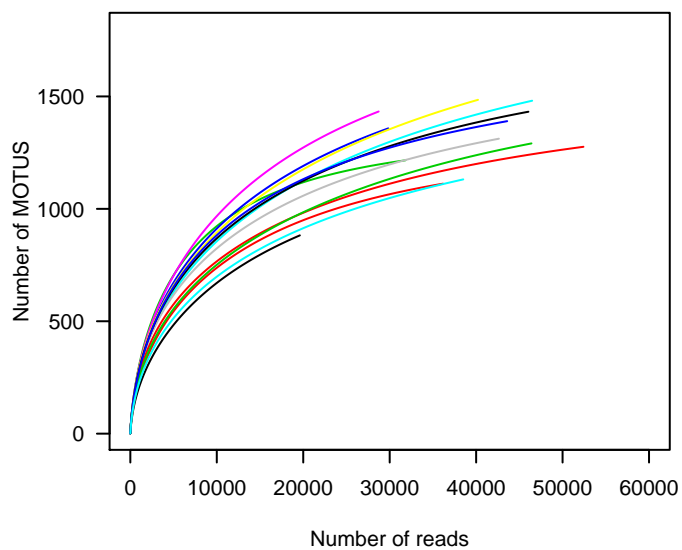

**Jamaica COI**

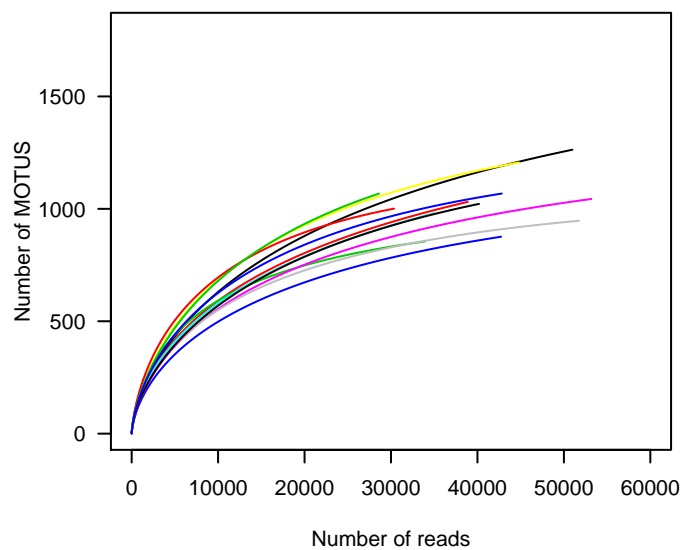

**Turks & Caicos COI**

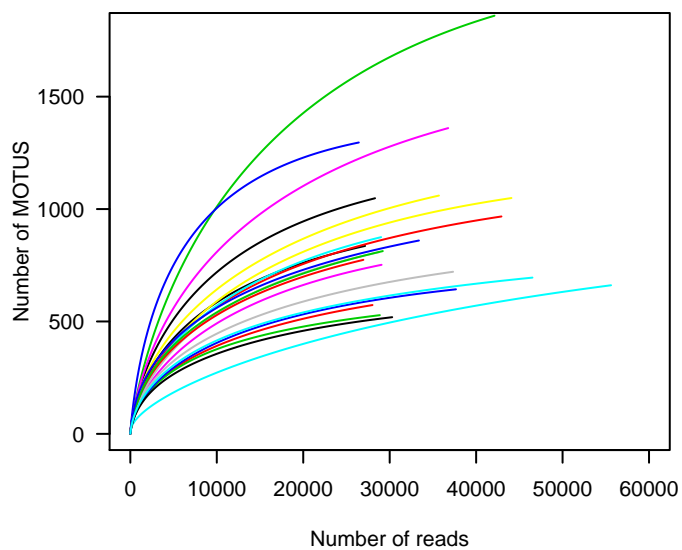

Supplement: Supplementary file 3 [file ECE3-9-14341-s003.pdf]

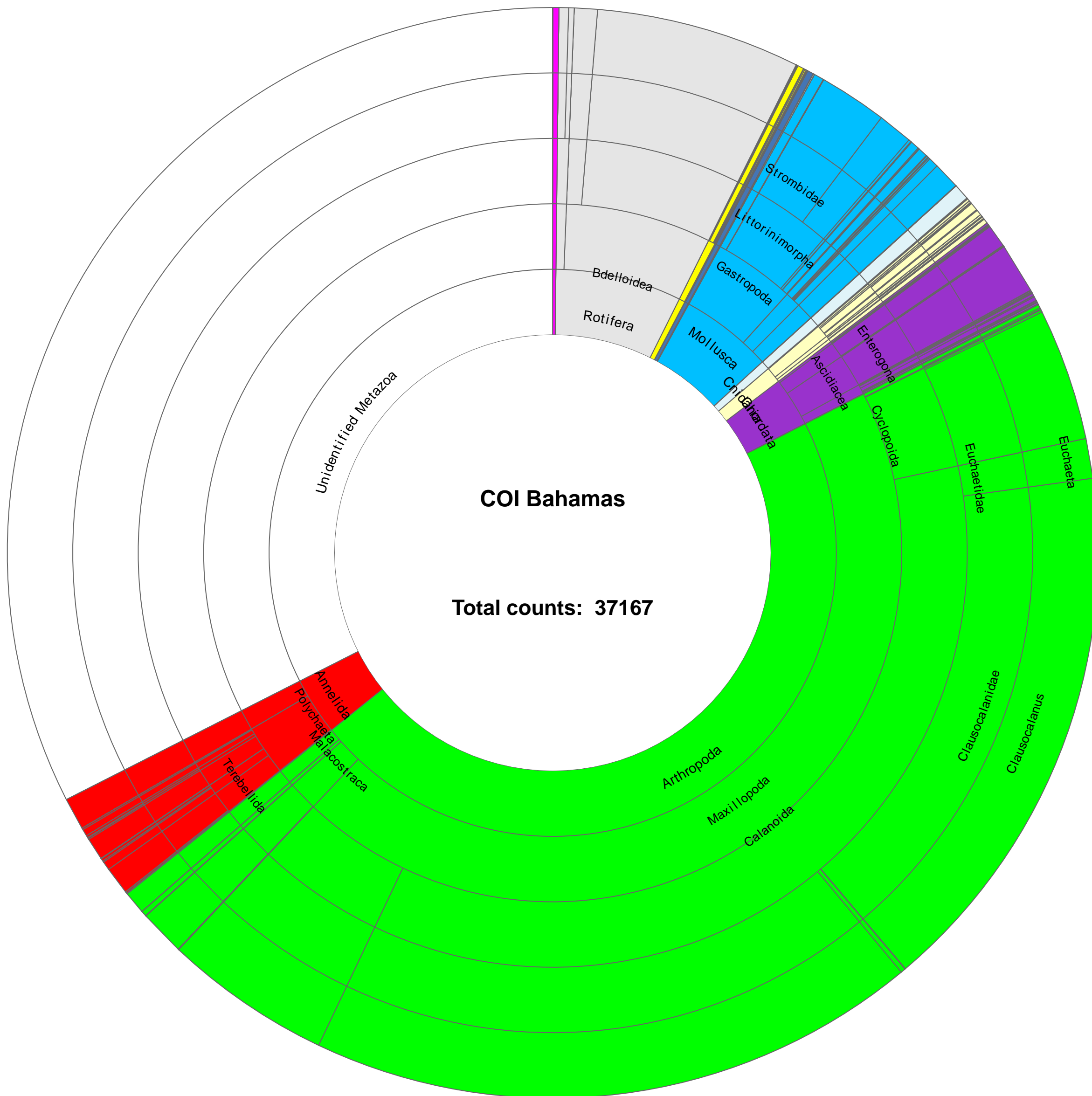

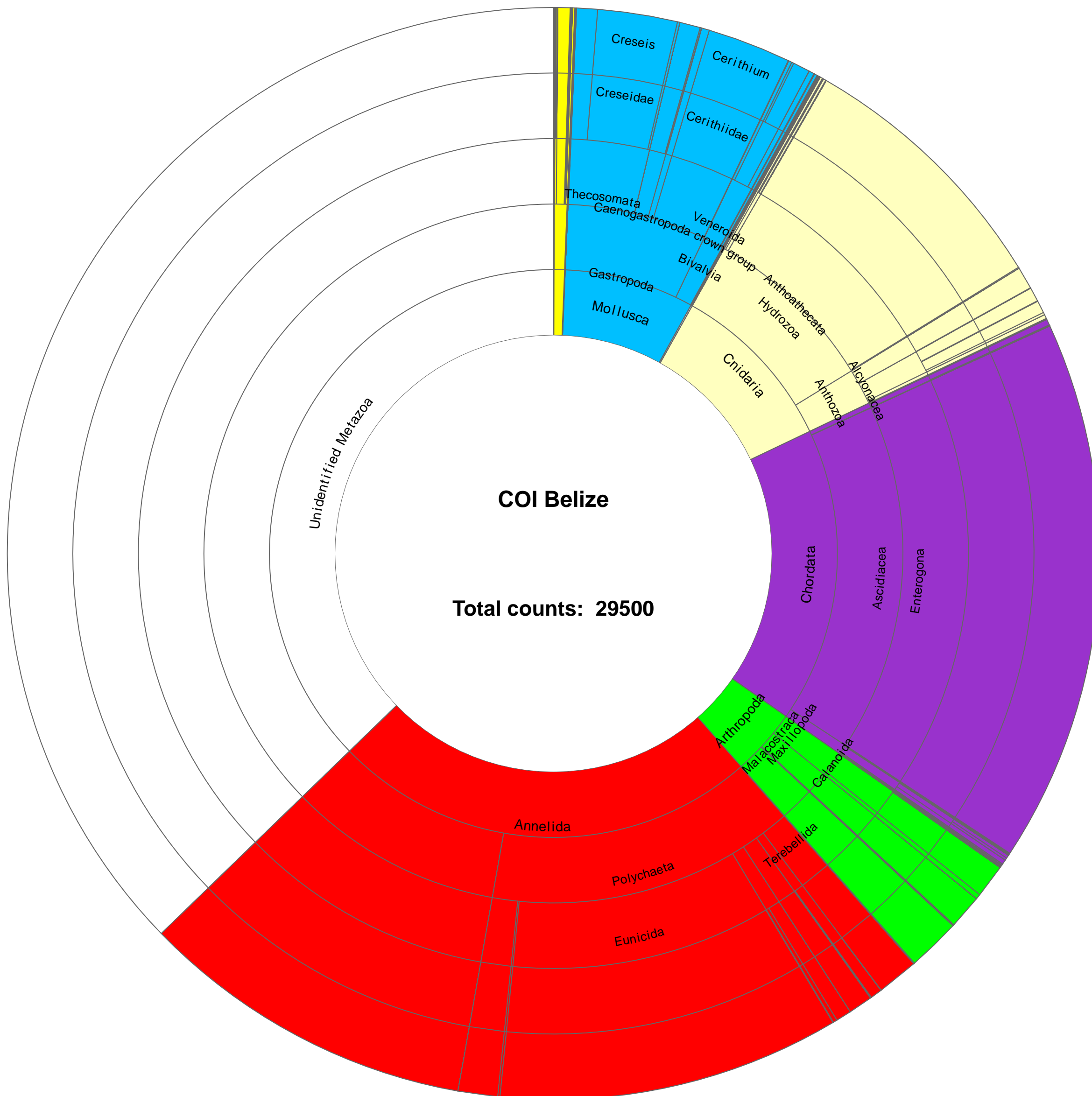

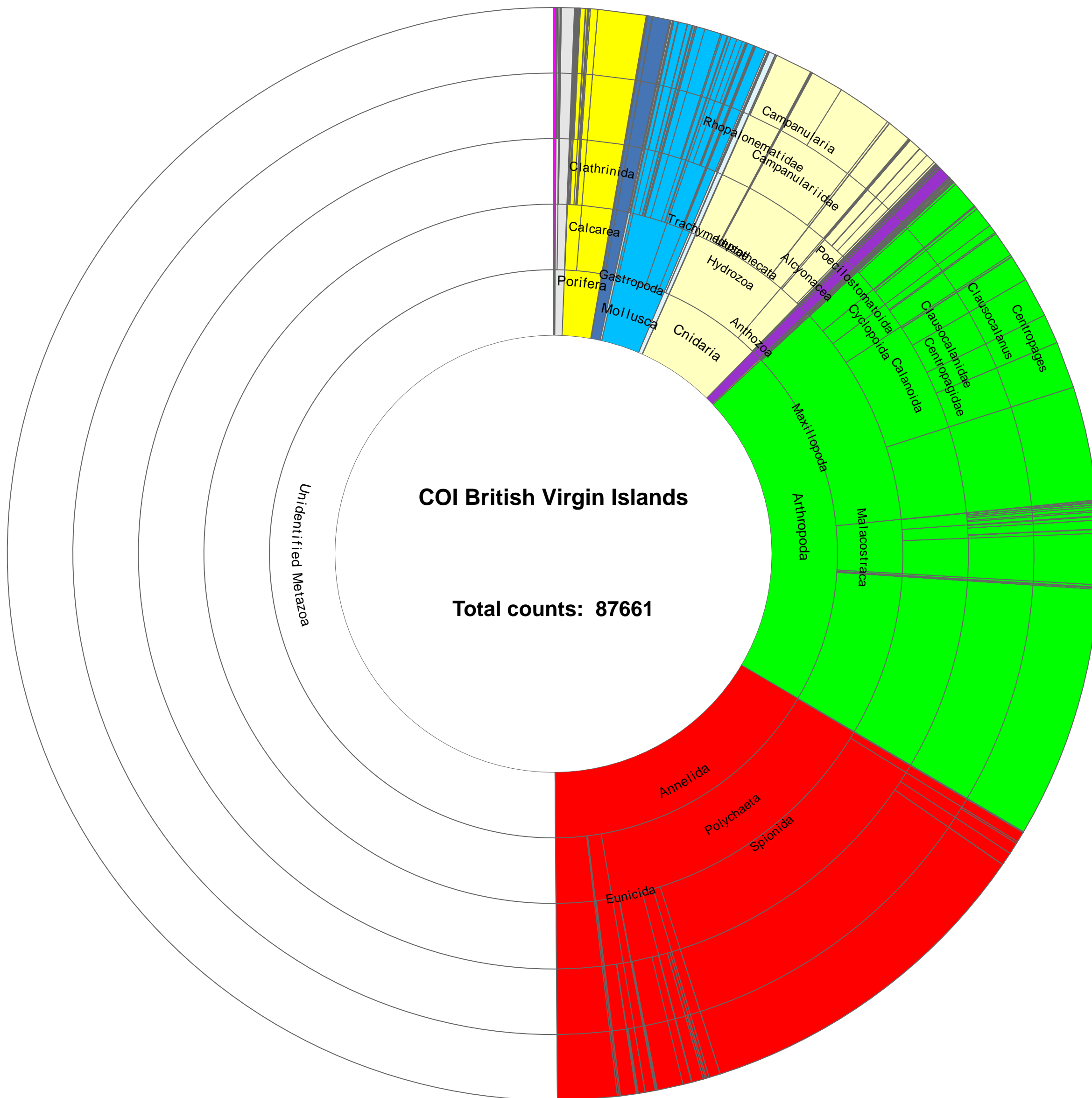

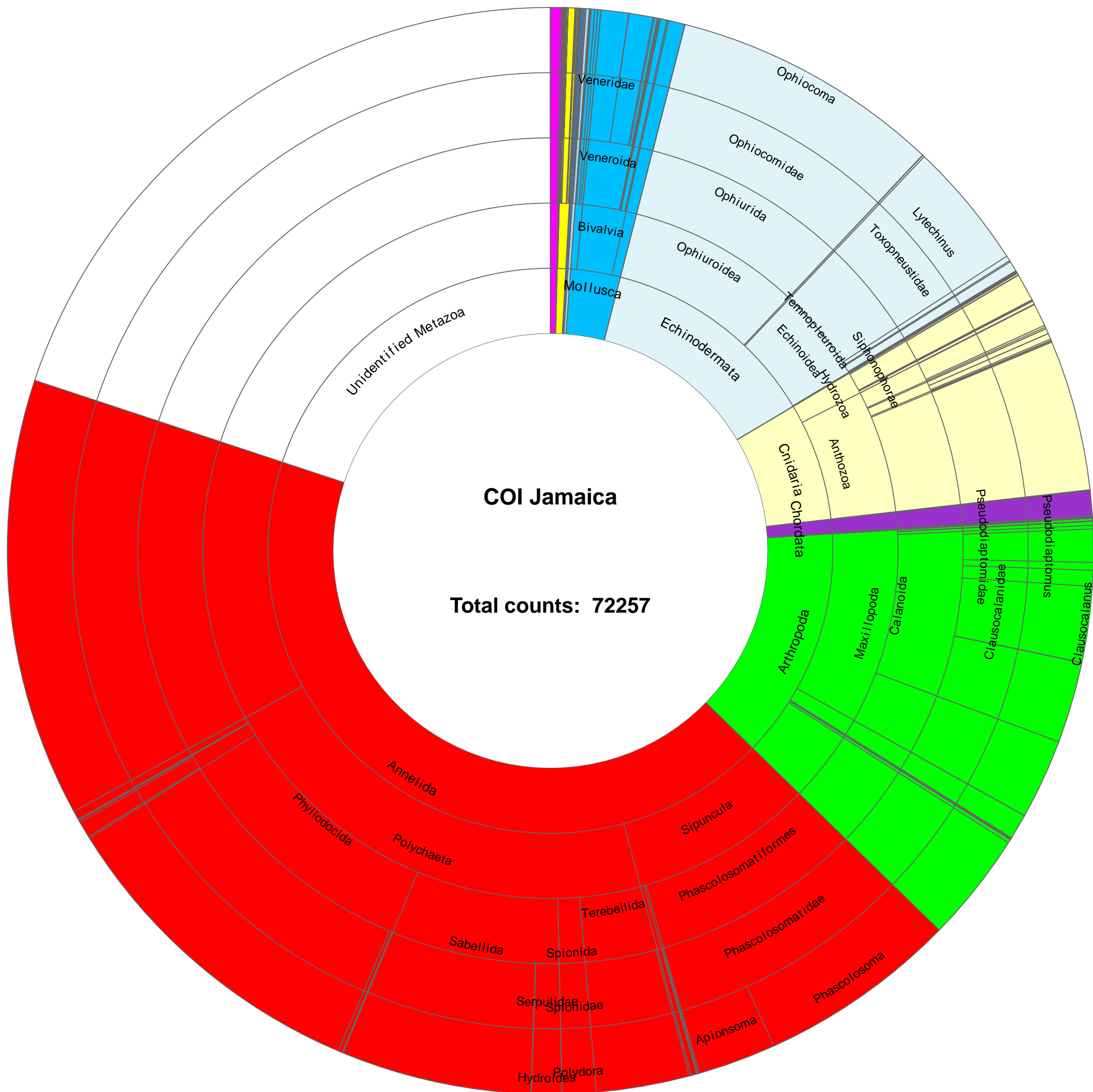

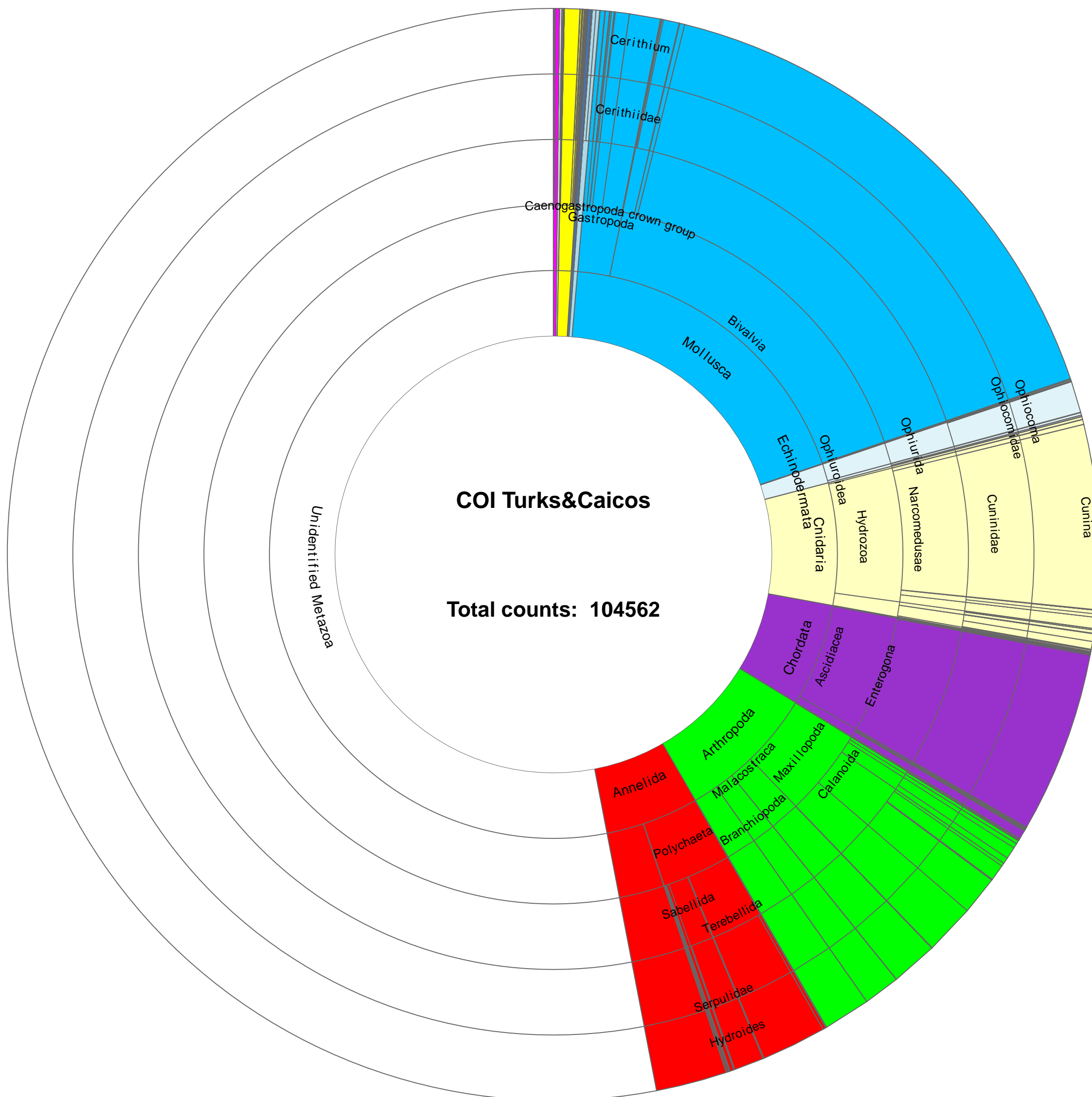

Supplement: Supplementary file 4 [file ECE3-9-14341-s004.pdf]
